# Deformation Behavior and Microstructural Evolution Coordinated Regulation by Compression Deformation for Metastable Ti Alloy

**DOI:** 10.3390/ma17246145

**Published:** 2024-12-16

**Authors:** Xueli Wang, Penglai Jia, Taoqin Wang, Fuguo Li, Qiang Wang

**Affiliations:** 1 School of Materials Science and Engineering, North University of China, Taiyuan 030051, China; 2 State Key Laboratory of Solidification Processing, School of Materials Science and Engineering, Northwestern Polytechnical University, Xi’an 710072, China

**Keywords:** metastable β titanium alloy, stress-induced martensite transformation, strain rate, martensitic twinning, microscopic morphology

## Abstract

In this paper, in order to investigate the harmonious relationship between the compression deformation behavior of metastable β titanium alloy and the microstructure evolution, the β solution-treated Ti-10V-2Fe-3Al (Ti-1023) alloy was compressed at room temperature and its deformation behavior was analyzed. Optical microscopy (OM) and field emission electron microscopy (FESEM) were used to study the microstructure evolution of alloys at different strain rates. The results show that the stress-induced martensite transformation (SIMT) is more easily activated by low strain rate compression deformation, which is conducive to improving its comprehensive mechanical properties. With the decrease in strain rate, the α″ martensite content increases significantly, the average grain size decreases substantially, and the Low Angle Grain Boundary (LAGB) volume fraction decreases correspondingly. In addition, after compression at different strain rates, the misorientation angle (MA) of the β matrix is mainly concentrated in the LAGBs. The change is small with the decrease in strain rate, but the α″ martensite orientation difference angle shows some peaks, which are ~60°, ~85°, and ~95°, respectively. Simultaneously, the strain rate has an important effect on the content and type of martensitic twins. Finally, the fracture morphology analysis shows that with the increase in strain rate, the fracture mode changes from ductile fracture to brittle fracture. The fracture surface presents a significantly elongated cavity along the direction of maximum shear stress.

## 1. Introduction

In the 1980s, the metastable β titanium alloy attracted researchers’ attention for the first time because of its good mechanical properties and corrosion resistance [1,2]. In recent years, due to the rapid development of aerospace, biomedicine, and other industries, people are constantly looking for new alloys with higher performance for application in such fields [3,4], and metastable β titanium alloys have once again attracted attention [5]. It is reported that the deformation mechanism of metastable β titanium alloy is closely related to strain rate, loading condition, initial grain size, and β phase stability [6,7]. Twin and martensite transformation plays an important role in the deformation process [8]. Therefore, it is pivotal to investigate the microstructure and mechanical behavior of metastable β titanium alloy during deformation to improve the comprehensive properties of the alloy.

The α″ martensite plays a key role in the mechanical properties of metastable β titanium alloy [9]. A better understanding of SIMT and the α″ martensite evolution is crucial to improve the properties of the alloy. At present, the deformation behavior of α″ martensite in metastable β titanium alloy is seldom reported. Generally, because of the self-regulation of the α″ martensite microstructure, the shear stress induced by the transformation of the variant itself is weakened in α″ martensite. The shape change in β matrix in SIMT is weakened [10,11,12]. Moreover, different types of twinning occur in a given β grain, such as {111}_α″_-type I, {211}_α″_-type II, {011}_α″_-compound, and {130}<3$\bar{1}$0>_α″_-deformation twinning [13,14,15]. Ping et al. [16] found that the ω phase and β phase in Ti-30Nb-3Pd were completely transformed into α″ with (110) α″ twin structure during room temperature deformation. Zhang et al. [17] suggested that metastable β Ti alloy with enhanced mechanical properties can be realized by α″ → β reverse martensite transformation and ω precipitation. Ma et al. studied Ti-10V-2Fe-3Al treated by β solution, in which the results show that the stress and yield strength are positively correlated with the strain rate. Meanwhile, the increment of the work hardening rate is negatively correlated with the strain rate [18]. Bobbili et al. [19] investigated the flow behavior of Ti-10-2-3 alloy at high strain rates and elevated temperatures using Split hopkinson tension bar (SHTB). And, the Johnson–Cook(J-C) material model was used to predict the flow behavior of Ti-10-2-3 alloy under these conditions.

However, the dependence of the synergistic effect of SIMT and twinning on the strain rate in metastable β titanium alloy has been not systematically understood. Therefore, in order to understand the mechanism of α″ martensite transformation and the twinning structure of α″ martensite in different orientations, we carried out the following: in this paper, metastable β titanium alloy (Ti-10V-2Fe-3Al) treated by β solution was selected to investigate the deformation behavior and microstructure evolution at compressive deformation with different strain rates. The work hardening rate, the evolution of α″ martensite and twinning, and the orientation relationship of α″ martensite and β matrix during deformation are discussed in detail.

## 2. Materials and Methods

**Figure 1:**
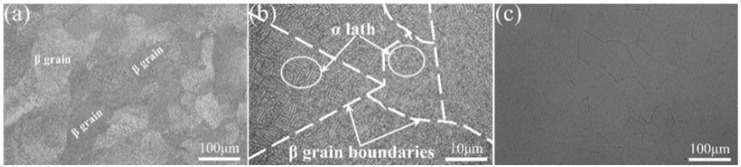
The representative microstructure of Ti-1023 alloy: (**a**,**b**) as-received sample; (**c**) β solution treatment sample.

**Figure 2:**
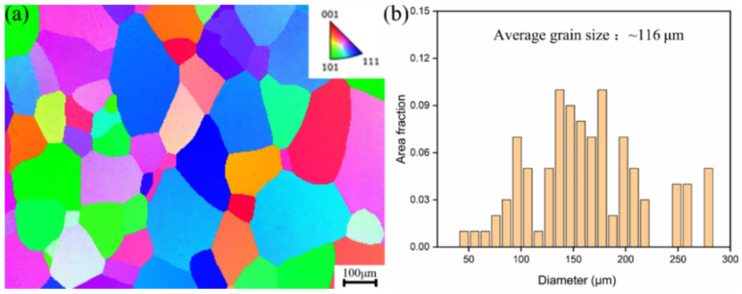
Metastable β-Ti-1023 alloy after β solution treatment: (**a**) the inverse pole figure (IPF); (**b**) corresponding grain size.

The Ti-10V-2Fe-3Al (Ti-1023) alloy is produced into bars with a diameter of 85 mm by hot forging. The chemical composition of Ti-1023 alloy is shown in Table 1 and measured by the Cypress Alloy Analyzer of SciAps X-200 (SciAps, Boston, MA, USA) purchased by Shenzhen Lai Lei Technology Development Corporation (Shenzhen, China). The initial microstructure of the alloy is composed of α phase and β matrix, as shown in Figure 1a. After β solution treatment at 840 °C for 30 min, the sample was rapidly quenched by water, and the β phase was completely restored to the initial state, as shown in Figure 1b. As shown in Figure 2, the microstructure information of the alloy was obtained by EBSD in this experiment, and the average grain size of the β phase was about 116 μm analyzed with the equivalent circle diameter method by the AZtecCrystal 2.1.2 software (AZtecCrystal, Oxford, UK).

**Figure 3:**
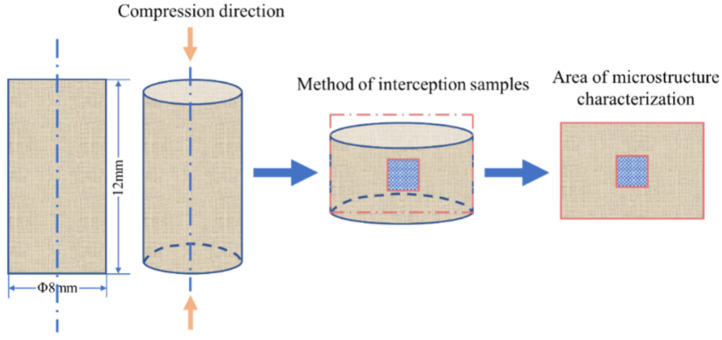
Schematic diagram of sample dimension and deformation for uniaxial compression test.

Uniaxial compression experiments were carried out by the INSTRON-3382 mechanical testing machine (INSTRON, Norwood, MA, USA). The compression behavior of metastable β titanium alloys at different strain rates was studied by quasi in situ test. The strain rates are 10^−1^ s^−1^, 10^−2^ s^−1^, 10^−3^ s^−1^, 10^−4^ s^−1^, and 10^−5^ s^−1^, respectively. Figure 3 shows the cylindrical specimen with a diameter of 8mm and a height of 12 mm, the axial compression deformation mode, and the microscopically characterized slices after deformation. The specimens were mechanically polished into a specular surface and subjected to 7 h vibration polishing. The microscopic characterization and fracture morphology observation were performed by field emission electron microscopy (FESEM, Merlin Compact, Zeiss, Oberkochen, Germany) to obtain the phase composition and grain orientation data of Ti-1023 alloy. At last, the data were post-processed by the AZtecCrystal 2.1.2 software (AZtecCrystal, Oxford, UK).

## 3. Results and Discussion

### 3.1. Analysis of Metastable β Phase Deformation Mechanism

**Figure 4:**
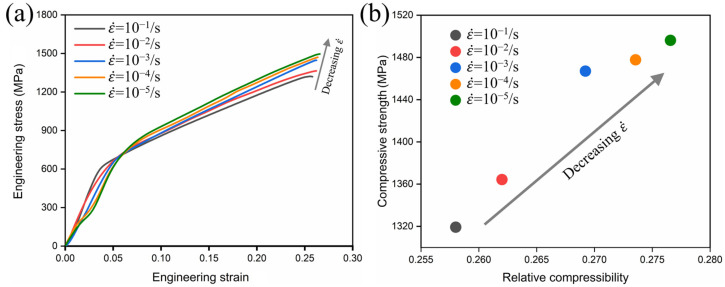
Compression with different strain rates: (**a**) engineering stress–strain curves; (**b**) relationship between compression strength and relative compressibility.

Figure 4 shows the engineering stress–strain curves of metastable β-Ti-1023 alloy under compression at different strain rates, as well as the relationship between compressive strength and relative compression ratio. As the compression strain rate decreases, both the compressive strength and relative compression ratio increase. And, the stress–strain curve gradually transitions from a single yield to a distinct double yield phenomenon, as shown in Figure 4a. The double yield phenomenon is a sign of SIMT activation. This indicates that the studied alloy is less likely to exhibit a double yield phenomenon at a high strain rate, but it is very obvious at a low strain rate. The occurrence of SIMT can effectively enhance the strength and toughness of the specimen, indicating that the alloy exhibits negative strain rate sensitivity (NSRS) after yielding during compression. This effect has also been observed and described in detail in other metastable β-Ti alloys [3,20]. It suggests that low strain rate compression deformation is more likely to activate SIMT so as to divide the β matrix into fine grains of different sizes which could achieve grain refinement strengthening. Additionally, during the compression process, the engineering stress–strain curve indicates that different stages of deformation correspond to different deformation mechanisms. They consist of the elastic–plastic deformation of the single β phase, the activation and development of SIMT, and the elastic-plastic deformation of the residual β phase and α″ martensite. Additionally, from Figure 4a,b, it can be observed that decreasing the strain rate from 10^−1^ s^−1^ to 10^−5^ s^−1^ leads to a gradual decrease in yield strength regardless of whether SIMT occurs. However, both compressive strength and relative compressibility exhibit a growing trend. The compressive strength and relative compressibility reach approximately 1496 MPa and 27.6% at a strain rate of 10^−5^ s^−1^. The above analysis indicates that low strain rate compression deformation is more conducive to improving the compressive strength and relative compressibility of the metastable β-Ti-1023 alloy.

**Figure 5:**
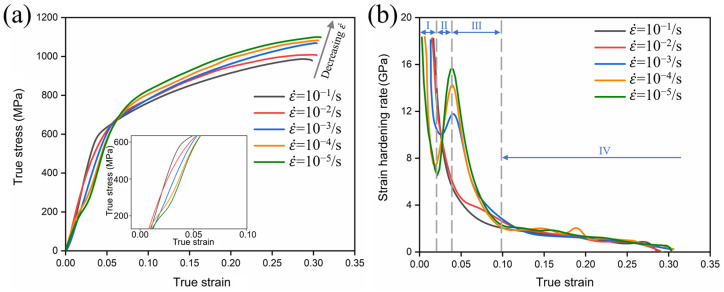
Compression deformation with different strain rates: (**a**) true stress–true strain curves; (**b**) strain hardening rate curves.

Figure 5 shows the true stress–true strain curve of metastable β-Ti-1023 alloy from compression and the evolution law of strain hardening rate (θ=∂σ/∂ε) with true strain. It can be seen from Figure 5a that the high strain rate compression deformation does not show the double yield phenomenon, while the low strain rate compression shows the obvious double yield phenomenon. And the lower the strain rate, the more obvious the double yield phenomenon. Figure 5b shows that the deformation hardening curves of $\dot{\varepsilon }$ = 10^−1^ s^−1^ and $\dot{\varepsilon }$ = 10^−2^ s^−1^ show display similar laws, while the deformation hardening curves from $\dot{\varepsilon }$ = 10^−3^ s^−1^ to $\dot{\varepsilon }$ = 10^−5^ s^−1^ show different rules. According to the analysis of compression deformation behavior, the strain hardening rate curve of the compression of the alloy includes four evolution stages, and different evolution stages correspond to the activation of different deformation mechanisms. It shows that the compression strain rate plays a very important role in the activation and evolution of various mechanisms during the plastic deformation process of metastable β-Ti-1023 alloy [21].

In view of the above phenomena, the influence of strain rate can be explained from the following two aspects: one is the activation and development of SIMT, in which the coherent interface between β phase and α″ martensite is formed to coordinate the lattice distortion, and the incoherent interface is formed to reduce the elastic strain energy [22]. Because the interface formed by SIMT activation will hinder the subsequent nucleation and growth of α″ martensite [18], the interface motion is also crucial to the nucleation and growth of α″ martensite. According to the dislocation model proposed by researchers based on the interface structure [23], the strain rate dependence of interface motion is similar to that of slip dislocation. With the decrease in strain rate, the resistance of dislocation motion decreases accordingly. Therefore, low strain rate deformation is more conducive to interface motion, which promotes SIMT and increases the content of α″ martensite in deformed samples. The other is the temperature effect in accordance with the motion theory model of thermally activated interface. The heat generated by the activation and development of SIMT in the sample cannot be released in time, leading to a higher temperature gradient when the sample is compressed at a high strain rate. And, the β phase is more stable at high temperatures. Consequently, under the condition of high strain rate compression deformation, SIMT is relatively difficult to sustain [10,21]. The amount of α″ martensite in high strain rate compression deformed specimens is relatively low.

### 3.2. Effect of Martensite Transformation on Grain Refinement Strengthening

**Figure 6:**
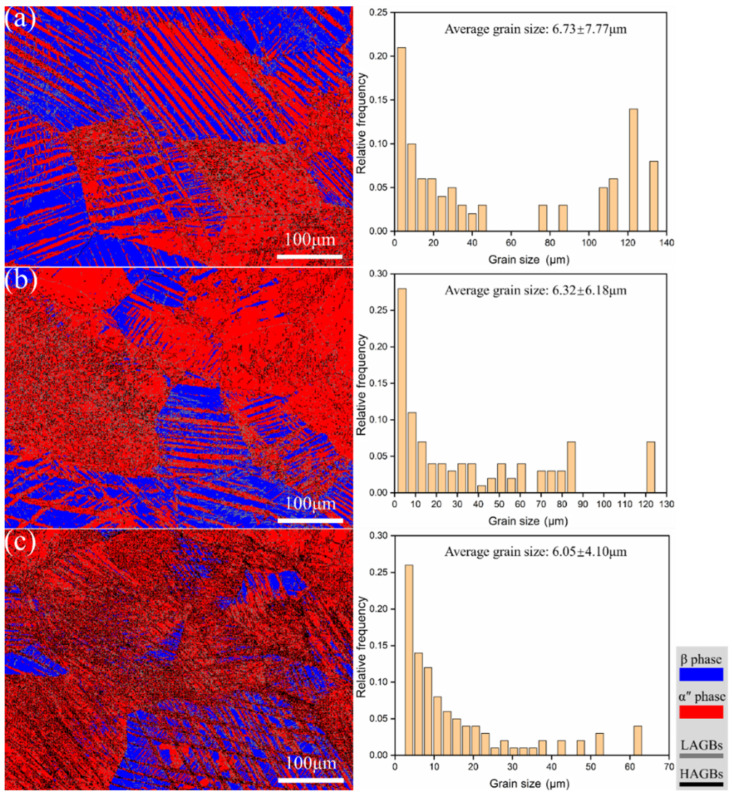
Phase constitution and grain size maps by compression deformation to fracture with different strain rates: (**a**) 10^−2^ s^−1^; (**b**) 10^−3^ s^−1^; (**c**) 10^−5^ s^−1^.

The phase distribution and grain size histograms of the metastable β-Ti-1023 alloy compressed to fracture at different strain rates are shown in Figure 6, where the phase and grain boundary composition percentages are shown in Table 2. The grain boundary angle is also another essential index for the microstructure of materials. The grain boundary angles can be divided into low-angle grain boundaries (LAGBs ≤ 15°) and high-angle grain boundaries (HAGBs > 15°), being noted by the gray lines and black lines in Figure 6. As shown in Figure 6a, the α″ martensite often initiates and grows at the grain boundaries of the β matrix and the α″ martensite, showing a strip-shaped and α″/α″ intersected massive structure. Likewise, part of the β matrix almost shows a complete α″ martensitic group, with a content of about 59.1%. Its average grain size is ~6.73 μm. In addition, the LAGB content is ~60.7%. It is shown that the SIMT is activated during the compressive deformation, resulting in significantly refined grains and a high content of LAGBs. When the strain rate is reduced to $\dot{\varepsilon }$ = 10^−3^ s^−1^, the α″ martensite still presents elongated and α″/α″ intersected blocky structure. Moreover, more α″ martensite groups are almost formed in the β matrix. Hence, the content of α″ martensite increases significantly to ~76.5%, and the average grain size decreases to ~6.32 μm. The content of LAGBs is also reduced to 56.6%, as shown in Figure 6b. When the strain rate decreases to $\dot{\varepsilon }$ = 10^−5^ s^−1^, the fine α″ martensite is still formed in the β matrix, and the amount of the original β matrix containing almost complete α″ martensite group increases significantly, which makes the content of α″ martensite relatively increase to ~85.7%. The average grain size was refined to about 6.05 μm, and the volume fraction of LAGB decreased to about 41.6% (Figure 6c).

It can be concluded that the amount of α″ martensite increases significantly and the average grain size decreases significantly with the decrease in strain rate. It indicates that the alloy shows compression rate sensitivity. And, that low strain rate compression is more conducive to the occurrence of SIMT. Along with the continuation of compression, it is conducive to the decomposition of β matrix and α″ martensite. Further, finer grains are formed, resulting in continuous grain refinement and significant grain refinement strengthening. In addition, the volume fraction of LAGBs increases with the increase in compressive deformation strain rate. It demonstrates that LAGBs are easy to produce during high strain rate compressive deformation, which provides more nucleation points for α″ martensite and promotes the occurrence of SIMT [24]. Further LAGBs are usually regarded as a series of lattice dislocations. It can hinder the grain growth and refine the grains significantly, resulting in fine grain strengthening, which indicates that the compressive strain rate has an important effect on the microstructure evolution of metastable β-Ti-1023 alloy.

**Figure 7:**
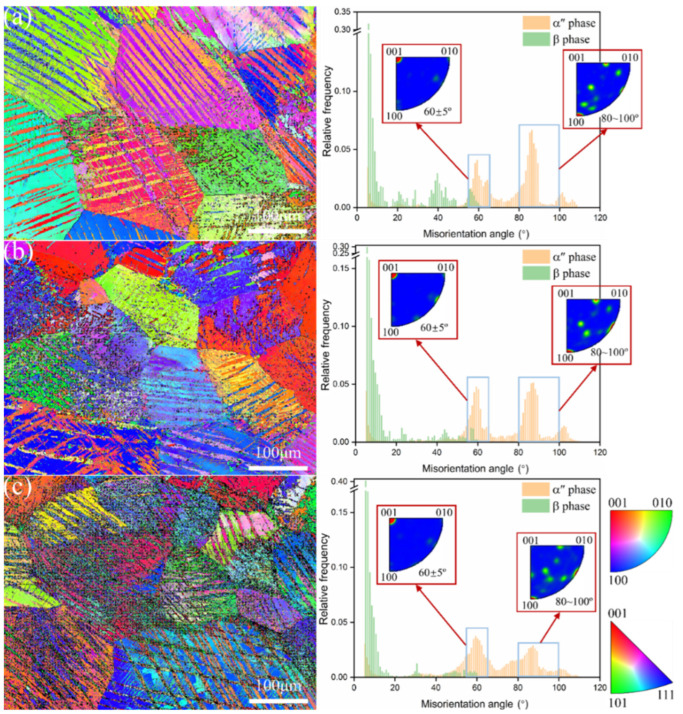
IPF image and misorientation angle distribution by compression deformation to fracture with different strain rates: (**a**) 10^−2^ s^−1^; (**b**) 10^−3^ s^−1^; (**c**) 10^−5^ s^−1^.

Figure 7 shows the IPF and misorientation angle distribution of metastable β-Ti-1023 alloy compressed to fracture at different strain rates, and different colors in the figure represent different grain orientations [25]. It can be seen from Figure 7a that during compression deformation at $\dot{\varepsilon }$ = 10^−2^ s^−1^, strip-shaped or α″/α″ intersecting blocky α″ martensite with different orientations will be generated on the β matrix. Due to the different orientations of the β phase, the SIMT in different β matrixes after applying compressive stress is also different, resulting in different content, morphology, and orientation of α″ martensite in each grain. With the decrease in compression strain rate, the β grains containing high content of α″ martensite group increase. And, the morphology and orientation of α″ martensite in different β matrixes are more diversified, but they still show long strips, α″/α″ intersection blocks, or laths (Figure 7b,c). In addition, some fine or thin needle-like α″ martensite structures will be embedded in the early α″ martensite at a strain rate of 10^−5^s^−1^, as shown in Figure 7c. The results show that a more complex heterostructure composed of β phase and α″ martensite group can be formed by low strain rate compression, and the strain rate has an important effect on the morphology and distribution of α″ martensite structure.

The percentage of misorientation angle also changes significantly with the decrease in strain rate. From the histogram of the misorientation angle in Figure 7, it is evident that the MA of the β matrix in the alloy after compression at different strain rates is mainly concentrated in low-angle grain boundaries (LAGBs). It changes slightly with the decrease in strain rate, but the misorientation angles of α″ martensite will show some peaks. They are located at ~60°, ~85°, and ~95°, respectively, exactly corresponding to the {111}_α″_-type I, {211}_α″_-type II, {011}_α″_-compound twinning, and {130}_α″_-deformation twins. The analysis results of each type of twin content are summarized in Table 3, indicating that different degrees of DIMT are activated by compression deformation at different strain rates. The reason is that the coalescence of the α″ variants during deformation is hindered by dislocations pinned at the grain boundaries of the variant interactions, which eventually deform within each α″ variant in the form of martensite twins [13,15,26]. Therefore, the activation and development of compression morphing DIMT at different rates will be analyzed next.

**Figure 8:**
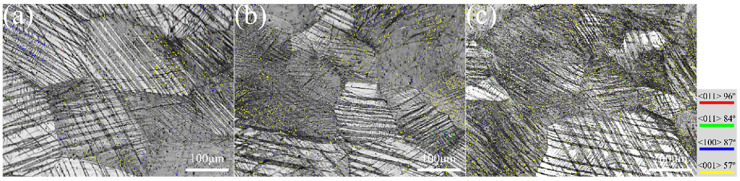
Twin boundary distribution by compression deformation to fracture with different strain rates: (**a**) 10^−2^ s^−1^; (**b**) 10^−3^ s^−1^; (**c**) 10^−5^ s^−1^.

Figure 8 shows the evolution of the distribution of different types of twin boundaries with decreasing strain rates. As can be seen from Figure 8, martensite twins mainly nucleate at the grain boundaries of the α″ martensite group. Then, it grows and passes through the lath and slender α″ martensite microstructure or ends in the grain. Finally, various types of martensite twins with different orientations are formed [25]. At a strain rate of 10^−2^ s^−1^, it can also be seen from Figure 8 and Table 3 that the content of {111}_α″_-type I and {211}_α″_-type II twins is relatively low and can be ignored. And, the deformed samples are mainly composed of {011}_α″_-compound twinning and {130}<3$\bar{1}$0>_α″_-deformation twins, with little difference in content. The content of {011}_α″_ twins decreases, while the content of {130}<3$\bar{1}$0>_α″_ twins increases. The other two kinds of twins are still negligible at a strain rate of 10^−3^ s^−1^. And the content of {011}_α″_ twins continues to decrease, while the content of {130}<3$\bar{1}$0>_α″_ twins continue to increase. The content of the {111}_α″_ I and {211}_α″_ II twins is still negligible at a strain rate of 10^−5^ s^−1^. The results show that only low contents of {111}_α″_ I and {211}_α″_ II twins are generated during compression, while suggesting that low strain rate compression can promote the formation of {130}<3$\bar{1}$0>_α″_ twins and hinder the formation of {011}_α″_ twins. In fact, the formation of martensitic twin structure on α″ martensite contributes to grain refinement and strain hardening. At the same time, the formation of martensitic twin structures can effectively reduce the rapid strain localization caused by twin formation, leading to improved strength and plasticity simultaneously [22]. Therefore, the detailed analysis of the special orientation relationships (SORs) between β matrix and α″ martensite is helpful in revealing the effects of compression at different rates on the activation and development of SIMT and DIMT in metastable β-Ti-1023 alloy.

### 3.3. Influence of Strain Rate on Martensite Orientation

**Figure 9:**
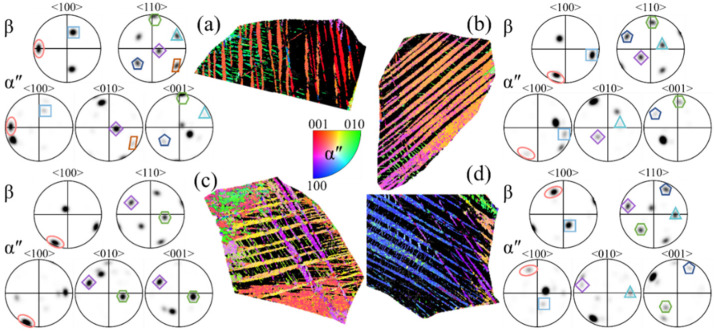
The SORs of β matrix and α″ martensite variants by compression deformation to fracture at $\dot{\varepsilon }$ = 10^−2^ s^−1^: (**a**–**d**) the grains with representative morphology of α″ martensite.

**Figure 10:**
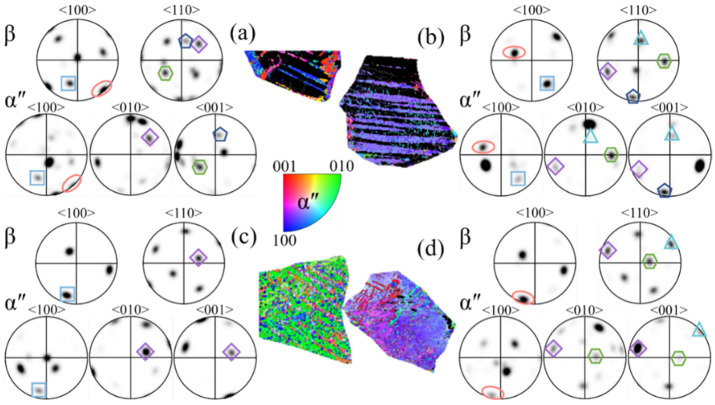
The SORs of β matrix and α″ martensite variants by compression deformation to fracture at $\dot{\varepsilon }$ = 10^−3^ s^−1^: (**a**–**d**) the grains with representative morphology of α″ martensite.

**Figure 11:**
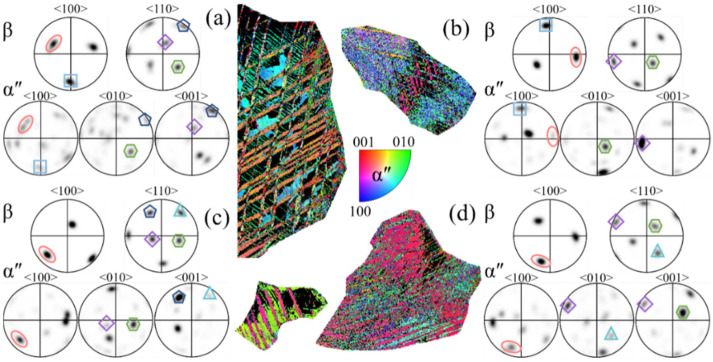
The SORs of the β matrix and α″ martensite variants by compression deformation to fracture at $\dot{\varepsilon }$ = 10^−5^ s^−1^: (**a**–**d**) the grains with the representative morphology of α″ martensite.

Representative grains containing the typical morphologies of α″ martensite were extracted from Figure 7, showing the SORs between the β matrix and α″ martensite of the metastable β-Ti-1023 alloy at different strain rates in Figure 9, Figure 10 and Figure 11. At a strain rate of 10^−2^ s^−1^, it can be seen from Figure 9a that many kinds of α″ variants formed in the β matrix, but there are five types of SORs between the β matrix and the α″ variants. In Figure 9b,d, there are four types of SORs between the β matrix and the α″ variants. In Figure 9c, the orientations of the α″ variants in the β matrix are more diversified, while only two of the β matrix and α″ variants satisfy SORs. The results show that there are SORs between the lath or α″/α″ massive martensite and the β matrix, and the growth direction of the α″ variants in different orientations in the β matrix is different. The reason is that the activation energy required for the SIMT activation of initial β grains with different orientations is different from that required for the growth of the α″ variants in the same β grains, which leads to the difference between the β matrix and the α″ variant species formed inside it.

At a strain rate of 10^−3^ s^−1^, it is observed that there are three types of SORs between the β matrix and α″ variant in Figure 10a,d, four types in Figure 10b, and only one kind in Figure 10c, respectively. As the strain rate decreases, although the content of α″ martensite increases, the types of SORs between the β matrix and α″ variant do not increase. It is suspected that the formation of more martensite twins makes the orientation of the α″ variants more diversified. At a strain rate of 10^−5^ s^−1^, it can be seen from Figure 11a,d that there are three kinds of SORs between the β matrix and the α″ variant, two kinds of SORs between the β matrix and the α″ variant in Figure 11b, and four kinds of SORS between the β matrix and the α″ variant in Figure 11c. As the strain rate continues to decrease, the number of SORs increases slightly due to the significant increase in the α″ martensite content.

The above results show that the strain rate of compression has a marked effect on the orientation relationship between the β matrix and α″ variant. The high strain rate of compressive deformation is more likely to induce some preferred orientations of α″ martensite along SORs and form a regular change in the orientation of α″ martensite. Inhomogeneous stress during compression deformation will lead to the continuous rotation of grains. As a result, adjacent grains which have different orientations, and voids or overlaps are easy to occur between grains [27]. Therefore, the accurate evaluation of GNDs is helpful to better understand the regular changes in the orientation relationship between the β matrix and α″ variants.

### 3.4. Relationship Between Dislocation Density and Strain Rate

**Figure 12:**
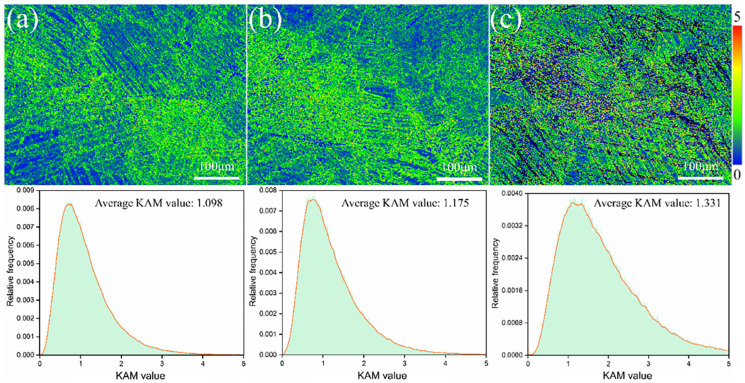
KAM distribution maps by compression deformation to fracture with different strain rates: (**a**) $\dot{\varepsilon }$ = 10^−2^ s^−1^; (**b**) $\dot{\varepsilon }$ = 10^−3^ s^−1^; (**c**) $\dot{\varepsilon }$ = 10^−5^ s^−1^.

Figure 12 shows the KAM distribution and evolution diagram of metastable β-Ti-1023 alloy from compression deformation to fracture at different strain rates. Among them, KAM mainly represents the local misorientation between the β matrix and α″ martensite. And, the larger the KAM value, the larger the local misorientation. It can be seen from Figure 12 that as the compressive strain rate decreases (from $\dot{\varepsilon }$ = 10^−2^ s^−1^ to $\dot{\varepsilon }$ = 10^−5^ s^−1^), the KAM value increases, with the average value increasing from ~1.098 to ~1.175 and then to ~1.331. Due to the inhomogeneity of compression deformation, α″ martensite presents an uneven distribution. And a large number of α″ martensite groups formed in the microstructure, resulting in an inhomogeneous distribution of misorientation gradient. At a strain rate of 10^−2^ s^−1^, Figure 12a shows a large misorientation gradient from the β/α″ interface to the interior of α″ martensite in the deformed sample; that is, the maximum misorientation gradient occurs in the interior of α″ martensite. The β/β interface also produces some local misorientation, but the misorientation gradient is relatively small compared with the β/α″ interface. In addition, the relative content of the smaller KAM value is higher, and the maximum KAM value is about 3.5.

At a strain rate of 10^−3^ s^−1^, a conclusion similar to the above description can be drawn (Figure 12b), the relative content of the smaller value of KAM is slightly reduced, and the maximum value of KAM is about 4.2. At a strain rate of 10^−5^ s^−1^, Figure 12c shows the overall KAM value of the deformed specimen increases, reaching five. And, the area of the KAM maximum value region increases significantly. It is worth noting that the α″ martensite initiates and grows at the β matrix grain boundaries. So, each initial β grain is adjacent to at least one α″ martensite with a misorientation gradient (Figure 12). And, the more α″ martensite around the initial β grain, the more diverse the misorientation gradient in the deformed sample. The gradient of misorientation can be seen in all the sizes of α″ martensite, indicating that even a small size of α″ martensite can cause severe local plastic deformation. The size of KAM can truly reflect the level of GNDs; that is, the region with larger KAM must correspond to the region with higher GNDs.

**Figure 13:**
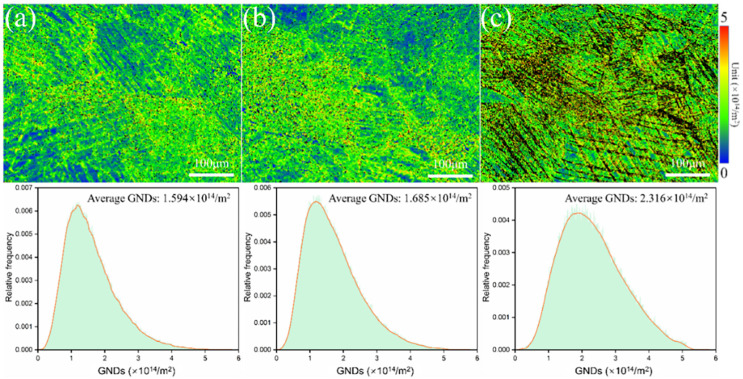
GND distribution maps by compression deformation to fracture with different strain rates: (**a**) $\dot{\varepsilon }$ = 10^−2^ s^−1^; (**b**) $\dot{\varepsilon }$ = 10^−3^ s^−1^; (**c**) $\dot{\varepsilon }$ = 10^−5^ s^−1^.

Figure 13 shows the distribution of the GNDs of metastable β-Ti-1023 alloy during compression deformation at different strain rates. As shown in Figure 13, the GNDs in the deformed specimen are distributed unevenly. With the decrease in the strain rate, the distribution area of the high GNDs area gradually expands. The average value of GNDs also increases from 1.594 × 10^14^/m^2^ to 1.685 × 10^14^/m^2^, and then to 2.316 × 10^14^/m^2^. As shown in Figure 13, the GNDs in α″ martensite are distinctly higher than that in β matrix because α″ martensite tends to show higher lattice distortion during deformation [28]. Therefore, the higher content of α″ martensite in the low strain rate deformation sample leads to a very high GNDs accumulation area in the sample, and the GND distribution gradient is more significant.

The above analysis shows that the increase in SIMT content can hinder dislocation movement and produce high dislocation density, which in turn provides nucleation sites for α″ martensite. This contributes to high strain hardening behavior [29]. It can be concluded that SIMT formation and dislocation accumulation promote each other [30]. Moreover, α″ martensite is mainly nucleated and expanded at parent phase grain boundaries and various crystal defects (such as dislocations) to generate high strain energy. Similarly, adjacent grain boundaries, phase boundaries, or twin boundaries are usually the preferred sites for GND aggregation and nucleation. Dislocation promotes plastic deformation, and the strain energy can be reduced so that dislocations can also provide sites for α″ martensite nucleation to eliminate certain defects or reduce the nucleation energy.

### 3.5. Fracture Characteristics

**Figure 14:**
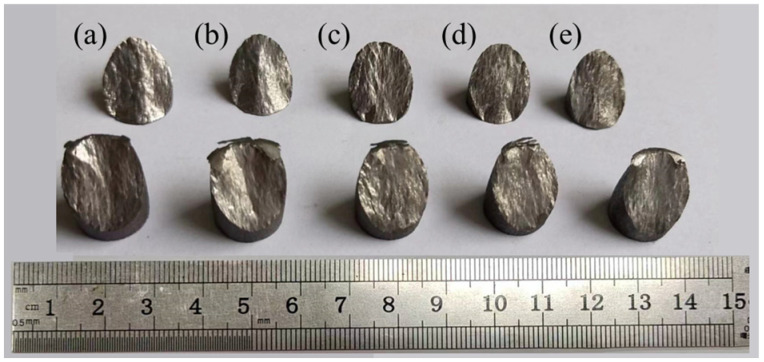
Macroscopic observation of fracture morphology during compression deformation with different strain rates: (**a**) $\dot{\varepsilon }$ = 10^−5^ s^−1^; (**b**) $\dot{\varepsilon }$ = 10^−4^ s^−1^; (**c**) $\dot{\varepsilon }$ = 10^−3^ s^−1^; (**d**) $\dot{\varepsilon }$ = 10^−2^ s^−1^; (**e**) $\dot{\varepsilon }$ = 10^−1^ s^−1^.

Fracture morphology analysis is an important means to infer the fracture mechanism of material [31]. In order to explore the intrinsic fracture mechanism of compression of the studied materials, Figure 14 shows the macroscopic fracture morphology of metastable β-Ti-1023 alloy at different strain rates. Regardless of the strain rate, all the fracture forms are inclined fracture surfaces along the direction of maximum shear stress, which is approximately 45° from the direction of compressive load, as shown in Figure 14. It suggests that the maximum shear stress plays a key role in the formation of compressive fracture, and the fracture surface always develops along the direction of the maximum shear stress. A general conclusion can be drawn that the compression fracture of the metastable β-Ti-1023 alloy is caused by the shear process.

**Figure 15:**
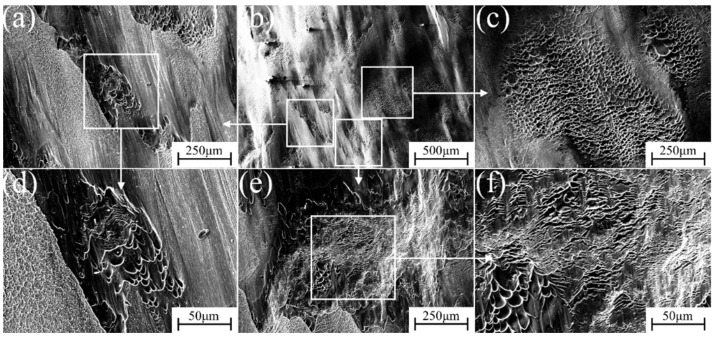
Fracture morphology during compression deformation at $\dot{\varepsilon }$ = 10^−5^ s^−1^: (**b**) low-magnification image of the fracture; (**a**,**c**,**e**) are the high magnification of (**b**); (**d**,**f**) are the further blowup of (**a**,**e**).

**Figure 16:**
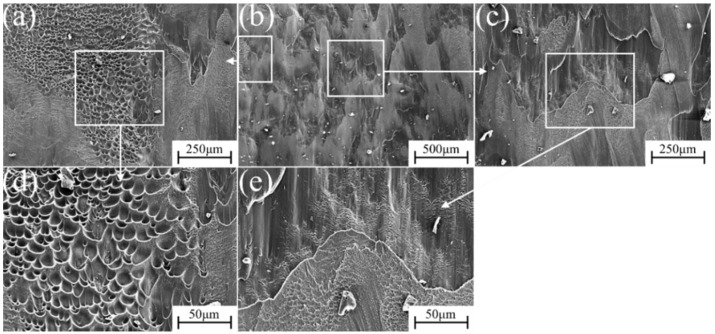
Fracture morphology during compression deformation at $\dot{\varepsilon }$ = 10^−4^ s^−1^: (**b**) low-magnification image of the fracture; (**a**,**c**) are the high magnification of (**b**); (**d**,**e**) are the further blowup of (**a**,**c**).

**Figure 17:**
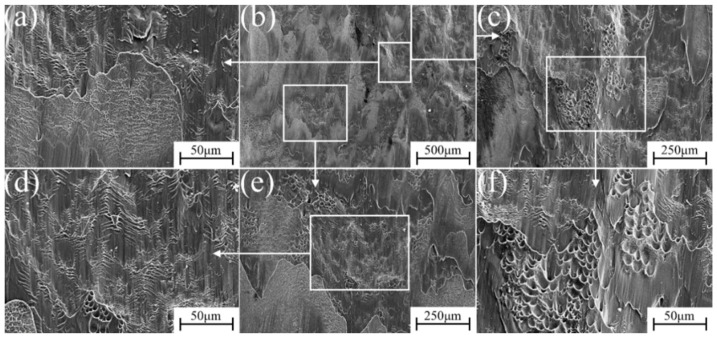
Fracture morphology during compression deformation at $\dot{\varepsilon }$ = 10^−3^ s^−1^: (**b**) low-magnification image of the fracture; (**a**,**c**,**e**) are the high magnification of (**b**); (**d**,**f**) are the further blowup of (**c**,**e**).

**Figure 18:**
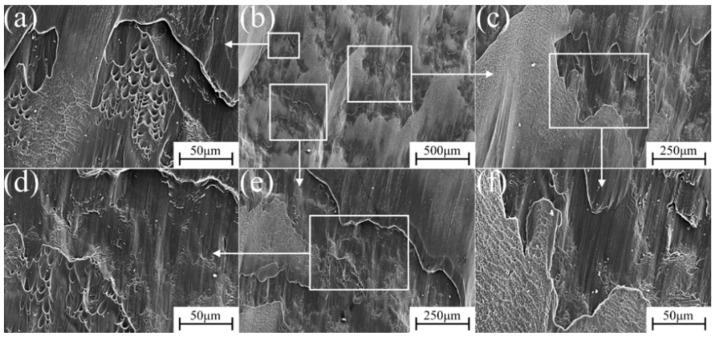
Fracture morphology during compression deformation at $\dot{\varepsilon }$ = 10^−2^ s^−1^: (**b**) low-magnification image of the fracture; (**a**,**c**,**e**) are the high magnification of (**b**); (**d**,**f**) are the further blowup of (**c**,**e**).

**Figure 19:**
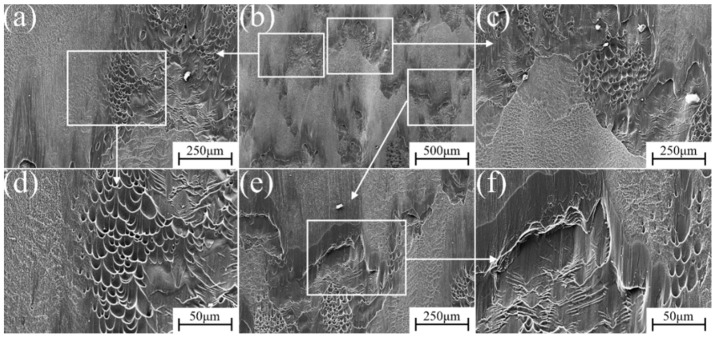
Fracture morphology during compression deformation at $\dot{\varepsilon }$ = 10^−1^ s^−1^: (**b**) low-magnification image of the fracture; (**a**,**c**,**e**) are the high magnification of (**b**); (**d**,**f**) are the further blowup of (**a**,**e**).

The micro-fracture morphology of α″ martensite influenced by different strain rate compression deformation in metastable β-Ti-1023 alloy is shown in Figure 15, Figure 16, Figure 17, Figure 18 and Figure 19. At a strain rate of 10^−5^ s^−1^, the fracture surface is not smooth. It is suspected that it is caused by a large number of micro-dimples with small intervals on the fracture surface [32]. There are also numerous dimples in the fracture morphology. They are deep and elongated in the shear direction. Furthermore, there are some brittle fracture features in fracture morphology, namely the cleavage plane, as shown in Figure 15. With the strain rate of 10^−4^ s^−1^, more lamellar structures can be found in the fracture morphology. The cleavage plane area is larger than before, and the fracture tends to be smooth gradually. The pit area decreases and becomes shallow, but there are also plenty of small dimples accumulated in the pit (Figure 16). At a strain rate of 10^−3^ s^−1^, the area of the cleavage plane on the microscopic fracture morphology increases, and the fracture surface tends to be flat. Accordingly, the number and size of dimples decrease significantly, indicating that the brittle fracture characteristics are more obvious as is exhibited in Figure 17.

At a strain rate of 10^−2^ s^−1^, the area of the cleavage plane on the microscopic fracture surface continues to increase. The fracture surface becomes more flat and gradually close to the plane. In addition, small steps have been formed in some areas, which shows that the brittle fracture characteristics of the deformed specimen are increasing, as shown in Figure 18. At a strain rate of 10^−1^ s^−1^, Figure 19 shows that the fracture surface of the specimen seems to have a cleavage fracture. The small steps formed in some areas become deeper, and only a few stacked dimples can be observed in the fracture morphology. The cleavage plane occupies most of the area, indicating that the brittle fracture characteristics are dominant at this time.

Comparing Figure 15, Figure 16, Figure 17, Figure 18 and Figure 19, with the increase in strain rate, the fracture mode changes from ductile fracture to brittle fracture, which is related to the content of α″ ductile phase, as shown in Figure 6 and Table 2 above. The fracture surface shows obvious elongated cavities along the direction of the maximum shear stress. It signifies that the compressive strain rate has a significant impact on the fracture morphology and the fracture mechanism. During compression deformation, metastable β-Ti-1023 alloy produces SIMT and DIMT under compressive stress. As a result, it makes the grain size significantly decrease and produces a fine-grained strengthening effect. Correspondingly, the defect size in fine-grained materials is small. At the same time, the activation and accumulation of GNDs promote the strain strengthening of the samples. Therefore, the refinement and strain-strengthening ability of low strain rate deformation is stronger. Ergo, the energy required for fracture is higher [33].

Owing to the significant influence of the plastic deformation process on the grain boundary characteristics, LAGBs and HAGBs with different volume fractions can be produced at different strain rates. During the propagation of cleavage cracks, they continuously pass through the grain boundary and converge to form a large cleavage plane. When the crack intersects with the LAGBs, it can constantly pass through the grain boundary, and the cleavage plane no longer increases [32] (Figure 17e). When the crack passes through the ordinary HAGBs, a large number of cleavage planes will appear due to the large misorientation between adjacent grains, and the altitude intercept of cleavage steps is also considerable (Figure 19e). Meanwhile, dislocation motion is usually hindered at grain boundaries, twin boundaries, or phase interfaces, resulting in dislocation pile-up zones, where microcracks nucleate [32]. When the microcrack propagation encounters the α″ ductile phase, the crack tip is easily blunted. It causes the crack to change direction or branch so as to relax the energy [32]. Therefore, the overall effect of stress-induced martensite transformation is to improve the toughness of the material. And, low strain rate deformation is more conducive to improving the toughness of metastable β-Ti-1023 alloy.

## 4. Conclusions

The effects of different compressive strain rates on the deformation behavior and microstructure evolution of metastable β-Ti-1023 alloy were systematically studied. The results enrich the mechanism of α″ martensite transformation and α″ martensite twin structures with different orientations, and have certain guiding significance for the study of the strengthening and toughening mechanism of this alloy. The main conclusions are drawn as follows:

1. As the increase in strain rate, the double yielding phenomenon caused by martensitic transformation is not obvious, which is due to the stronger tendency of SIMT activation and development under the condition of low strain rate compression deformation. In addition, the compression deformation of metastable β-Ti-1023 alloy at a low strain rate is more conducive to improving its comprehensive mechanical properties.
2. During compression deformation, because SIMT is activated at a low strain rate, more β matrix is transformed into α″ martensite and decomposed, resulting in fine grain strengthening. In addition, the proportion of each twin is different in the compression process with different strain rates, which promotes the formation of {130}<3$\bar{1}$0>_α″_ deformation twins and hinders the formation of {011}_α″_ combination twins at low strain rates. The formation of martensite twins reduces the localization of rapid strain and improves strength and toughness.
3. High strain rate compression deformation is more likely to induce α″ martensite to produce some preferred orientations that comply with SORs, and forms regular changes in α″ martensite orientation. The average values of KAM and GNDs increase significantly with the decrease in strain rate, and the increase in α″ martensite content will produce higher dislocation density due to the continuous activation of SIMT. There is a gradient distribution of dislocation density in all the sizes of α″ martensite, and the grain boundary of α″ martensite can effectively hinder the dislocation movement and produce high strain hardening ability.
4. The fracture mode is mainly ductile fracture under low strain rate deformation, and there are a large number of deep and large dimples and a small number of smooth cleavage planes in the fracture surface. Under high strain rate deformation, the brittle fracture characteristics are dominant, the fracture surface is more flatter and close to the plane, the number and size of dimples are significantly reduced, and the area of the smooth cleavage plane is significantly increased.

## Figures and Tables

**Table 1:** The main chemical composition (wt.%) of the as-received Ti-1023 alloy.

Element	V	Al	Fe	Si	N	H	O	Ti
Weights/%	10.59	3.70	1.70	0.09	0.005	0.001	0.042	balance

**Table 2:** The volume fractions of phases and grain boundaries after compression deformation with different strain rates.

Strain Rate (s^−1^)		$\dot{\varepsilon }$ = 10^−2^	$\dot{\varepsilon }$ = 10^−3^	$\dot{\varepsilon }$ = 10^−5^
Phase composition (%)	β phase	40.9	23.5	14.3
	α″ phase	59.1	76.5	85.7
Grain boundary composition (%)	LAGBs	60.7	56.6	41.6
	HAGBs	39.3	43.4	58.4

**Table 3:** The volume fractions twin boundaries after compression deformation with different strain rates.

	Proportion (%)	Deformation Rate (s^−1^)		
Twinning Types		$\dot{\varepsilon }$ = 10^−2^	$\dot{\varepsilon }$ = 10^−3^	$\dot{\varepsilon }$ = 10^−5^
{111}_α″_ I Type		0.27	0.10	0.22
{211}_α″_ II Type		0.70	0.88	0.44
{011}_α″_ Compound		14.0	5.59	2.22
$\{130\}<3\bar{1}0>$_α″_ Deformation		12.4	26.5	31.6

## Data Availability

The original contributions presented in the study are included in the article, further inquiries can be directed to the corresponding author.

## References

[1] Hanada S., Izumi O. (1987). Correlation of tensile properties, deformation modes, and phase stability in commercial β-phase titanium alloys. Metall. Mater. Trans. A.

[2] Hanada S., Izumi O. (1986). Transmission electron microscopic observations of mechanical twinning in metastable beta titanium alloys. Metall. Mater. Trans. A.

[3] Chen C.Y., Lu C.X., Yang X.J., Jing J.R., Huang X., Li J.S., Lai M.J. (2024). Remarkable contribution of stress-induced martensitic transformation to strain-hardening behavior in Ti−Mo-based metastable β-titanium alloys. Scr. Mater..

[4] Guan X., Liu D., Qu S., Cao G., Wang H., Feng A., Chen D. (2024). Multiple Deformation Mechanisms in Adiabatic Shear Bands of a Titanium Alloy during High Strain Rate Deformation. Materials.

[5] Naseri R., Casillas G., Mitchell D.R.G., Savvakin D.G., Ahmed M., Furuhara T., Pereloma E.V. (2021). Effect of strain on microstructural development during uniaxial compression of metastable beta Ti–10V–2Fe–3Al alloy. Mater. Sci. Eng. A.

[6] Ahmed M., Wexler D., Casillas G., Ivasishin O.M., Pereloma E.V. (2015). The influence of β phase stability on deformation mode and compressive mechanical properties of Ti–10V–3Fe–3Al alloy. Acta Mater..

[7] Shang G., Gan X., Wang X., Ge J., Li C., Zhu Z., Zhang X., Zhou K. (2024). Effect of Cooling Rate on α Variant Selection and Microstructure Evolution in TB17 Titanium Alloy. Materials.

[8] Bertrand E., Castany P., Yang Y., Menou E., Gloriant T. (2016). Deformation twinning in the full-α″ martensitic Ti–25Ta–20Nb shape memory alloy. Acta Mater..

[9] Gao P., Fan J., Sun F., Cheng J., Li L., Tang B., Kou H., Li J. (2019). Crystallography and asymmetry of tensile and compressive stress-induced martensitic transformation in metastable β titanium alloy Ti–7Mo–3Nb–3Cr–3Al. J. Alloys Compd..

[10] Chen N., Kou H., Wu Z., Qiang F., Wang C., Li J., Molina-Aldareguia J.M. (2021). Stress-induced α″ martensitic phase transformation and martensitic twinning in a metastable β titanium alloy. J. Alloys Compd..

[11] Ma X., Chen Z., Xiao L., Luo S., Lu W. (2021). Stress-induced martensitic transformation in a β-solution treated Ti–10V–2Fe–3Al alloy during compressive deformation. Mater. Sci. Eng. A.

[12] Zafari A., Xia K. (2018). Stress induced martensitic transformation in metastable β Ti-5Al-5Mo-5V-3Cr alloy: Triggering stress and interaction with deformation bands. Mater. Sci. Eng. A.

[13] Ji X., Gutierrez-Urrutia I., Emura S., Liu T., Hara T., Min X., Ping D., Tsuchiya K. (2019). Twinning behavior of orthorhombic-α″ martensite in a Ti-7.5Mo alloy. Sci. Technol. Adv. Mater..

[14] Lee H.J., Kim J.H., Park C.H., Hong J.-K., Yeom J.-T., Lee T., Lee S.W. (2023). Twinning-induced Plasticity Mechanism of α″-martensitic Titanium Alloy. Acta Mater..

[15] Zhang X., Wang W., Sun J., Gao Y., Pennycook S.J. (2022). Enhanced twinning-induced plasticity effect by novel {315}α″/{332}β correlated deformation twins in a Ti-Nb alloy. Int. J. Plast..

[16] Ping D., Cui C., Yin F., Yamabemitarai Y. (2006). TEM investigations on martensite in a Ti–Nb-based shape memory alloy. Scr. Mater..

[17] Zhang X., Wang S., Wu J., Sun J., Gao Y., He B., Pennycook S.J. (2024). ω-Strengthened Ti-23Nb alloy with twinning-induced plasticity developed via reverse martensitic transformation. Acta Mater..

[18] Ma X., Li F., Cao J., Sun Z., Wan Q., Li J., Yuan Z. (2017). Study on the deformation behavior of β phase in Ti–10V–2Fe–3Al alloy by micro-indentation. J. Alloys Compd..

[19] Bobbili R., Madhu V. (2016). Effect of strain rate and stress triaxiality on tensile behavior of Titanium alloy Ti-10-2-3 at elevated temperatures. Mater. Sci. Eng. A.

[20] Niessen F., Gazder A.A., Mitchell D.R.G., Pereloma E.V. (2021). In-situ observation of nucleation, growth and interaction of deformation-induced α″ martensite in metastable Ti–10V–2Fe–3Al. Mater. Sci. Eng. A.

[21] Suryawanshi J., Singh G., Msolli S., Jhon M.H., Ramamurty U. (2021). Tension-compression asymmetry and shear strength of titanium alloys. Acta Mater..

[22] Ashby M.F. (2006). The deformation of plastically non-homogeneous materials. Philos. Mag. A J. Theor. Exp. Appl. Phys..

[23] Castany P., Yang Y., Bertrand E., Gloriant T. (2016). Reversion of a Parent {130}⟨310⟩α″ Martensitic Twinning System at the Origin of {332}⟨113⟩β Twins Observed in Metastable β Titanium Alloys. Phys. Rev. Lett..

[24] Salem A.A., Kalidindi S.R., Doherty R.D. (2003). Strain hardening of titanium: Role of deformation twinning. Acta Mater..

[25] Cai M.-H., Lee C.-Y., Lee Y.-K. (2012). Effect of grain size on tensile properties of fine-grained metastable β titanium alloys fabricated by stress-induced martensite and its reverse transformations. Scr. Mater..

[26] Lai M.J., Tasan C.C., Raabe D. (2016). On the mechanism of {332} twinning in metastable β titanium alloys. Acta Mater..

[27] Calcagnotto M., Ponge D., Demir E., Raabe D. (2010). Orientation gradients and geometrically necessary dislocations in ultrafine grained dual-phase steels studied by 2D and 3D EBSD. Mater. Sci. Eng. A.

[28] Inamura T., Kim J.I., Kim H.Y., Hosoda H., Wakashima K., Miyazaki S. (2007). Composition dependent crystallography ofα″-martensite in Ti–Nb-based β-titanium alloy. Philos. Mag..

[29] Kundu A., Field D.P. (2016). Influence of plastic deformation heterogeneity on development of geometrically necessary dislocation density in dual phase steel. Mater. Sci. Eng. A.

[30] Heima A., Shinohara Y., Akamine H., Nishida M., Inamura T. (2024). Peculiar martensitic microstructure and dislocation accumulation behavior in Ti-Ni-Cu shape memory alloys almost satisfying the triplet condition. Acta Mater..

[31] Bantounas I., Dye D., Lindley T.C. (2010). The role of microtexture on the faceted fracture morphology in Ti–6Al–4V subjected to high-cycle fatigue. Acta Mater..

[32] Wang X., Li F., Xu T., Ma X., Hou B., Luo L., Liu B. (2021). Microstructure and microhardness evolution of Ti-10V-2Fe-3Al alloy under tensile/torsional deformation modes. J. Alloys Compd..

[33] Deng H., Liu J., Kang H., Guo Y., Song L., Yu H. (2025). Very high cycle fatigue behavior of TC4 titanium alloy: Faceting cracking mechanism and life prediction based on dislocation characterization. Int. J. Fatigue.

